# Genome Sequences of *Metabacillus* sp. Strain B2-18, Isolated from Human Skin, and Metabacillus endolithicus KCTC 33579^T^ 

**DOI:** 10.1128/mra.01348-22

**Published:** 2023-01-18

**Authors:** Haelim Son, Kyoung Lee

**Affiliations:** a Department of Bio Health Science, Changwon National University, Changwon, Republic of Korea; University of Rochester School of Medicine and Dentistry

## Abstract

*Metabacillus* sp. strain B2-18 was isolated from human skin. Here, we present the whole-genome sequences of this strain and Metabacillus endolithicus KCTC 33579^T^. The genomes of these strains contain 14 and 15 rRNA gene operons, respectively.

## ANNOUNCEMENT

In this study, we report the whole-genome sequences of *Metabacillus* sp. strain B2-18 and Metabacillus endolithicus KCTC 33579^T^ (1) to confirm the phylogenetic relationship between the two strains. A swab sample from the forehead skin of a female college student was directly streaked on minimal salts basal (MSB) agar (2) containing 0.5% yeast extract (MSBY medium). The agar medium was anaerobically incubated for 1 week at 28°C for growth. The anaerobic conditions were maintained in a tightly sealed jar with an Anaerobag (Chongqing Pang Tong, China). One isolate, B2-18, which was capable of microaerobic growth, was further purified by streaking on MSBY medium and aerobic incubation at 28°C for 3 days. This strain was deposited as KCTC 43380 (JCM 35039) and was assigned NCBI taxonomy identification number NCBI:txid2897333. Ethical approval for subject sampling was granted by the institutional review board of Changwon National University. Sanger sequencing of the 16S rRNA gene amplicon with primers 27F (5′-AGAGTTTGATCCTGGCTCAG-3′) and 1492R (5′-GGTTACCTTGTTACGACTT-3′) (3) yielded unidentifiable overlapping peaks with partially recognizable nucleotide sequences at the 5′ and 3′ ends. The amplification conditions were the same as those described previously (4). The resulting partial nucleotide sequences were used for homology analysis with the NCBI database and enabled the identification of B2-18 as belonging to the genus *Metabacillus*. The nucleotide sequences of the 16S rRNA gene of B2-18 from genome sequencing were deposited in GenBank (Table 1).

**TABLE 1:** General features of the genome sequencing and assembly

Feature	Finding for:	
	*Metabacillus* sp. strain B2-18	*M. endolithicus* KCTC 33579^T^
Isolation source	Human skin (South Korea)	Beach pebbles (India)
Sequencing analysis		
Illumina sequencing		
No. of reads	18,837,790	—^*a*^
Total read length (Gbp)	5.7	
Nanopore sequencing		
No. of reads	71,335	69,927
Total read length (Mbp)	748.9	366.2
Read length (mean ± SD) (bp)	10,499 ± 15,744	5,236 ± 7,852
Read *N*_50_ (bp)	28,156	10,667
Assembly analysis		
No. of contigs	2 circular	1 linear, 1 circular
No. of chromosomes and plasmids	1 chromosome, 1 plasmid	1 chromosome (linear, incomplete), 1 plasmid
Size (bp)	5,469,024	5,297,881
GC content (%)	35.5	35.5
Genome coverage (fold)	1,276.0	69.5
No. of protein-coding genes	5,090	4,660
No. of rRNA genes (5S, 16S, 23S)	15, 14, 14	16, 15, 15
No. of tRNA genes	148	146
No. of ncRNA genes^*b*^	8	7
No. of pseudogenes (total)	161	585
Estimated completeness (%)	99.12	98.11
Estimated contamination (%)	5.44	4.67
Data availability		
GenBank accession no. for genome assembly	ASM2111727v1	ASM2307833v1
SRA accession no. for raw sequencing data		
Illumina sequencing	SRX13239291	
MinION sequencing	SRX13239292	SRX14810261
GenBank accession no. for 16S rRNA genes	OP975733–OP975746	OP975759–OP975773

a —, not sequenced.

b ncRNA, noncoding RNA.

For DNA extraction, cells were cultured in a flask of MSBY broth for 48 h at 28°C, with shaking at 140 rpm. Total genomic DNA was purified using the phenol extraction method (5). Genomic DNA was sequenced with Illumina and Oxford Nanopore Technologies MinION platforms. Illumina sequencing was performed on a NovaSeq 6000 sequencer at DNALink Co. (Seoul, South Korea) as described previously (6). The whole-genome sequencing was performed using a TruSeq DNA PCR-free 550-bp library preparation kit (Illumina) and demultiplexing with bcl2fastaq2 v2.20 on the Illumina NovaSeq 6000 sequencer. The quality of the raw sequencing data was checked using FastQC (ASCII Q score offset, 33) (https://www.bioinformatics.babraham.ac.uk/projects/fastqc) (7). The read length was 2 × 151 bp (paired-end reads), with an insert size of approximately 550 bp, and the mean Phred quality score was 35.47. For Nanopore sequencing, libraries were prepared with purified raw DNA and the SQK-LSK109 kit (Nanopore) and multiplexed using the EXP-NBD104 barcoding kit according to the protocol. Sequencing was performed on a MinION sequencer using a FLO-MIN106 flow cell (R9.4.1) and MinKNOW v20.10.3 software with default settings. Reads were base called and demultiplexed in fast mode with a minimum Q score of 8 and then were further base called and barcode trimmed in super-accurate mode.

For comparison, M. endolithicus KCTC 33579^T^ obtained from the Korean Collection for Type Cultures was cultivated, and genomic DNA was purified under the same conditions as described for B2-18. The genomic DNA of KCTC 33579^T^ was sequenced with the Nanopore platform. The reads of strains B2-18 and KCTC 33579^T^ were assembled *de novo* using Unicycler with SPAdes v0.4.9b and Unicycler with miniasm v0.3, respectively, with default settings (8). The quality of the assembled genome sequences was evaluated using CheckM v1.1.3 (9). Default parameters were used for all software unless otherwise noted. Gene predictions and annotations were provided by NCBI using the best-placed reference protein set with GeneMarkS-2+ of the NCBI Prokaryotic Genome Annotation Pipeline (PGAP) v5.3 and v6.1 for B2-18 and KCTC 33579^T^, respectively (10).

Table 1 presents the general features of the genomes of B2-18 and KCTC 33579^T^. The assembly quality assurance report provided by NCBI showed that the best-matching type strain assembly for B2-18 is M. endolithicus KCTC 33579^T^ (GenBank assembly accession number GCA_023078335), with an ANI value of 95.40% and query coverage of 75.43%.

### Data availability.

GenBank accession numbers for raw sequencing data and genome assemblies are listed in Table 1.
